# What are the ‘necessary’ skills for a newly graduating RN? Results of an Australian survey

**DOI:** 10.1186/s12912-016-0144-8

**Published:** 2016-04-05

**Authors:** Roy A. Brown, Patrick A. Crookes

**Affiliations:** School of Nursing, Faculty of Science, Medicine and Health, University of Wollongong, Room 115, Building 41, Northfields Avenue, Gwynneville, NSW 2500 Australia; Honorary Appointment: School of Nursing, Faculty of Science, Medicine and Health, University of Wollongong, Wollongong, NSW Australia

**Keywords:** Competence, Skills, New graduate nurse

## Abstract

**Background:**

There appears to be a sense of disappointment with the product of contemporary nursing programs in Australia in that new graduate RNs are often referred to as not possessing appropriate skills by clinical colleagues. This work identifies the skills that the profession believes that newly graduating RN’s should possess at the point of registration.

**Methods:**

A qualitative consensus methodology was used in the form of a modified Delphi survey. Expert panels were used to review and validate data.

**Results:**

Consensus was reached on the top 25 skills areas that can be reasonably expected of a new graduate Registered Nurse in Australia. The top ranked skills areas included efficient and effective communication, professional nursing behaviours, privacy and dignity and managing medication administration.

**Conclusions:**

The consensus methodologies used to develop the skills areas indicated broad agreement across the profession in Australia. The complexity and context of practice was highlighted in the comments within the Delphi rounds. Interestingly no new skills were added and none removed from the initial list – some were prioritised over others but the majority agreed that all the skills areas were important for a newly graduating nurse.

## Background

The paper reports one aspect of a larger national study of competency assessment in nursing. The overall aim of the larger project funded by the Australian Learning and Teaching Council (ALTC) was to develop a competency assessment tool for nursing eligibility to practice programmes in Australia. The phase of the study reported in this paper sought to identify the ‘skills’ that the nursing profession could reasonably expect of a newly graduating registered nurse in Australia.

At the commencement of the project there were 39 universities delivering eligibility to practice programmes in Australia, the majority of these (*n* = 23) were located in metropolitan cities across Australia’s states and territories. There were a wide range and number of skills taught in nursing curriculum and as a consequence it was clear that the skills covered with preregistration nursing students were not consistent across programmes (Brown et al 2015 [1]; Crookes and Brown 2010 [2]). Since the introduction of national accreditation in 2010 course accreditation processes have been carried out by the Australian Nursing and Midwifery Accreditation Council (ANMAC); however there is no agreed consensus on the skill set that nursing students should possess or be developing during their programme of study. There is also no agreed consensus on the skill set required by industry that new graduate nurses should possess in Australia. In the literature it is identified that there is an observed difference between what is expected of a new graduate (a ‘work ready’ registered nurse), even though it is not defined, and the apparent end product of nursing registration programmes (Brown et al 2015 [1]; Burns & Poster 2008 [3]; Smith & Crawford 2003 [4]).

### Literature regarding the skill set of a newly registered nurse

A literature search and review to locate research on skills for newly graduated RNs identified over 500 papers, however much of this literature related to nurse practitioners or clinical nurse specialists or was in the form of discussion papers, literature reviews, government publications or textbooks.

Of the twenty publications finally identified relating specifically to new graduate RN skills, these primarily examined the practical or psychomotor skills required or frequently performed by new graduate RNs (Birks et al 2013 [5]; Burns & Poster 2008 [3]; Smith & Crawford 2003 [4]). These were all situated within acute medical/surgical settings. These articles were mainly descriptive studies, utilising surveys and Delphi methods, with descriptive approaches to analysis.

The literature also illustrates the tendency towards a transferability of terms where ‘skills’ and ‘competence’ are sometimes interchanged, this seems to stem from a lack of clarity regarding how performing a skill relates to the broader notion of competence. Greenberger et al 2005 [6] use terms such as ‘technical skill’ and ‘core skills’ undertaken within clinical course work as a means of differentiating ‘skills’ from ‘competence’ (Higgins et al (2010) [7], Clarke & Holmes (2007) [8]). Graduates view a lack of confidence in the performance of a skill with not being competent; this was reflected in Madjar et al’s (1997) [9] and Dufault (1990) [10] research; in these studies newly graduating registered nurses were competent in ‘problem solving’ for example but achieved lower scores related to completing ‘technical tasks’. There is a paucity of recent robust evidence in the area; and where evidence does exist it is based largely on self-report by the new graduate registered nurse (e.g. in Applin et al 2010 [11], Greenberger et al 2005 [6] and Boxer and Kluge 2000 [12]).

There is limited literature exploring perceptions of readiness, or competence; these are viewed from the perspective of the employer, for example the nurse manager (Oermann et al 2010 [13], Ramitru and Barnard 2001 [14]); experienced nurses; from the new graduate nurse (Duchscher (2009) [15], Boxer and Kluge 2000 [12]); or from patients (Calman 2006 [16]). Experienced RNs and nurse managers believe that new graduates would be ‘competent’ in practical nursing skills; so too did the new graduate, however the new RN did not feel competent in the more complex skills; the new RN believed that they would develop their competence more fully during their graduate year (Oermann et al 2010 [13]) or as Duchscher (2008) [17] termed this becoming during the ‘transition to registered nurse’.

A number of factors contribute to these perceptions such as: the duration of Bachelor of Nursing (BN) programmes (Candella and Bowles 2008 [18]); amount of clinical practice during the programme (fitness for practice, UKCC 1999 [19]); academic outcome differences (fitness for award), (Aiken et al 2014 [20], Aiken et al 2003 [21], Smith and Crawford 2003 [4]). This is compounded by literature that, on the one hand attempts to identify all the individual skills that nurses perform Alavi et al 1991 [22], Boxer and Kluge 2000 [12], Boxer et al 2001 [23] and Lee et al 2002 [24]) which is a rather reductionist strategy. Whereas on the other hand, an approach that attempts to define nursing in such broad terms such as an area of practice; that it renders the findings somewhat meaningless (*or maybe intangible*). Takase and Teraoka (2011) [25] developed the ‘Holistic Nursing Competency Scale’ and this, as well as Meretoja et al 2004’s [26] work on ‘indicators for competent nursing practice; illustrate the notion of the ‘distance between skills and competency’ and the ongoing attempts to connect them (Grugulis & Stoyanova 2011 [27], Payne 2000 [28], Hyland 1994 [29]). The research in this area has been almost exclusively on acute tertiary (hospital) care skills; with particular focus on aspects such as intravenous (IV) management (with a simple list of ‘bag’, ‘line’ and ‘site’ management); other areas such as hygiene needs are listed as a range of bathing techniques and mouth and denture care.

Nursing is a complex interplay of skill acquisition, competence development and increasing capability throughout the individual’s professional life (Benner 1984 [30], Benner et al 1996 [31], Eraut 1994 [32] and 1998 [33], Benner 2004 [34], Dreyfus and Dreyfus 1980 [35], Nursing and Midwifery Office, Queensland 2013 [36]). After initial registration the practitioner continues to further enhance and develop their practice within the workplace; inevitably the ‘starting point’ for a new graduate RN in terms of their capability and competence differs between graduates and so does the rate of ongoing development-illustrating the complexity of learning and application in nursing.

Boxer and Kluge (2000) [12] explored ‘essential nursing skills’ for a registered nurse in their first year of practice. The skills were viewed from the perspective of ‘which ones are frequently performed’ and if the skill was deemed to be ‘essential’ by the respondents, who were practicing newly registered nurses in a group of Australian metropolitan hospitals in one city. Boxer and Kluge (2000) [12] clearly identify this work as focussing on neophyte practitioners in acute medical and surgical settings.

Fourteen ‘skills’ were identified with up to twelve (12) subsets noted within each. Basic Life Support had no sub sets whereas Patient Nutrition had the greatest number of subsets (12); examples being ‘*assist/complete patient feeding*’ and ‘*insert intravenous cannulæ*’. The top 3 ‘most essential clinical skills’ as identified by the new registered nurses were; ‘disposal of sharps’; ‘hand washing’ and vital sign measurement’ and the lowest three were; ‘participate in group counselling’; ‘management of bladder irrigation’ and ‘continuous cardiac monitoring’. This work was replicated in 2001 (Boxer et al [23]) and included the views of ‘experienced nurses’. New graduates believed that ‘suction upper airway’, ‘managing gastric suction/drainage and ‘performing 12 lead ECG’s were the top three whereas experienced nurse said ‘removal of clips and sutures’, ‘collection of wound swab’ and ‘assist/complete patient feeding’. Whilst a detailed list of skills, this is a view of the ‘essential’ skills areas from an acute care perspective and does not consider the broad range of clinical settings in which a new graduate nurse may practice.

These skills are important to the nurse working within a hospital environment however in order to be able to provide, or supervise care given, the rather reductionist approach to the skills undertaken (Boxer et al (2001) [23], Boxer & Kluge (2000) [12]) is driven by what is perceived as ‘frequency of use’ rather than a range of skills that may be required on a somewhat infrequent basis but are also important, for example De-Escalation Techniques or Basic Life Support. This pragmatic ‘skills based’ approach is similar to Tollefson’s (2012) [37] text that many Australian universities have adopted; the text identifies 69 individual psychomotor skills. The text content clusters the skills under 15 headings (or Parts as termed by Tollefson) a few examples are: Aseptic technique (with 5 subset skills); Assessment (8); Medication Administration (8); Mental Health Skills (2); Observations (6); Personal Hygiene and Maintaining Skin Integrity (5); and Wound Management (4). This Australian text is widely used within Australian universities but there is no consensus on what skills are practiced and assessed across programmes in Australia.

Meretoja et al 2002 [38] and 2004 [26] illustrates the attempt at structuring nursing work into areas so that the ‘skills’ can be organised. Here are just two examples of ‘skills areas’ of the (74) items within the domains; firstly ‘supporting coping strategies’ within the ‘helping role’ domain and secondly ‘updating written guidelines for care’ within the ‘therapeutic interventions’ domain. Within these two examples the notion of a larger area – the ‘domain’ – are a sub set of skills areas.

Strategies such as this influenced the methodology employed within this study by avoiding reducing nursing to a list of skills; rather employing a strategy which reflected the complexity and the artistry of nursing practise. This strategy was supported by the expert group’s supporting the project; namely the Australian Nursing and Midwifery Council Professional Advisory Group (ANMC-PRG); the CDNM-ANZ as well as the project reference group. Thus the key goals in terms of developing the methodology were the avoidance of pursuing a reductionist strategy, acknowledging the key points from the literature review, which was on the whole focussed on acute tertiary care skills with relatively limited sample sizes and to broaden the sampling frame to ensure appropriate inclusion including; areas of clinical practice, rural and metropolitan areas and sectors, public, private and academic.

The use of Brown et al 2015 [1] audit of skills within curriculum documents in Australian universities was used as this utilised a methodology which ‘clustered’ a number of skills into what were termed ‘skills areas’. This provided a nationally already agreed structure for ‘skills areas’ to be used in the modified Delphi survey.

## Method

### Aim

This paper is drawn from a larger project which sought to develop a competency assessment schedule for use in eligibility to practice programmes in Australia Brown et al 2015 [1]; Crookes & Brown (2010) [2]. The main aim of this paper is to identify the necessary skills that can be reasonably expected of newly registered nurses.

### Design of the study

Inclusive, consensus methodologies were employed with the intention of maximising the contributions and ownership from the profession. The first step in achieving this was the development of a sampling frame in order to have a representative sample of the overall nursing population; academics, managers and clinicians from a range of clinical practice settings. Secondly, the skills areas audit data was utilised from Brown et al’s (2015)’s [1] audit of nursing programme curriculum documents in Australia and third consensus methods in the form of modified Delphi rounds were used to engage experts in the field to iteratively refine the skills areas list (Wilkes 2015 [39]; Asselin & Harper 2014 [40]; Hasson et al 2000 [41]; Delbecq, Van de Ven & Gustafson 1975 [42]).

### Participants

In order to maximise contributions from the profession local knowledge within states and territories was utilised by contacting Heads/Deans of Nursing Schools and Chief Nursing Officers. This local identification of individuals would assist in identifying local distribution networks for the survey links to be circulated. The Heads/Deans of nursing schools delivering RN programmes in Australia were obtained through the CDNM-ANZ, these universities were invited to participate and to identify an individual within their school to act as a liaison with the researcher (*n* = 39). The nominated individuals assisted in identifying local participants for the modified Delphi rounds. Secondly members of the groups of Chief Nursing Officers in each state and territory were asked to circulate the information through their contacts to reach as many members of the profession as possible (approximately 550 were approached through the contacts). These combined strategies were designed to maximise the number of survey respondents. Finally the ANMC PRG were invited to use their local networks to provide additional circulation lists for the survey. Each of these was sent a personal invitation, which included a participant information sheet concerning consent and explaining the purpose of the project/research and a link to the Survey Gizmo survey through their individual work emails.

### Data collection

An online survey tool was used in order to maximise the response rate and for ease of data handling (Survey Gizmo) to circulate the survey(s). In order to reduce ‘respondent fatigue’ bias the items were presented randomly to each participant (Lavrakas 2008 [43], Hart et al 2005 [44], Oppenheim 1992 [45]). The Delphi technique asks a group of experts to rank a list of items. Respondents in this modified Delphi were asked a variation of this, which was, ‘is x necessary’ or ‘is x not necessary’ then the scores were tallied and the items with highest scores remain for the next round. The modified Delphi approach allows respondents to contribute directly to the survey without being aware of other respondents’ contributions; this minimises any possible power differentials influencing respondent’s rankings. Prior to the ranking exercise a ‘predetermined cut off point’ was agreed – 80 %; then following the ranking exercise items below that point will be excluded from the next iteration/round. Usually two or three rounds are required to achieve consensus.

A qualitative comments section was also used to enable respondents to add new skills or to ‘explain/provide commentary’ why they have ranked particular items in certain ways.

### Data analysis

Online survey data was downloaded as Excel and Statistical Package for the Social Sciences (SPSS Version 19) files for analysis. The raw data and the qualitative comments were collated; the 80 % cut off criteria were applied then consideration was given in the light of the qualitative comments. The second modified Delphi was then undertaken by circulating the outcome of the initial round for validation with the same initial respondent group.

Qualitative comments were thematically analysed; following this they were then used to consider the implications of applying the agreed cut off criteria. For example many respondents ranked leadership as ‘not necessary’ but then suggested that leadership was important for inclusion within the skills areas in subsequent rounds of the modified Delphi – so they would be retained.

Finally expert panel members from the ALTC project management group, ANMC PRG and the CDNM were presented with the findings and discussed the analysis and explored the rationale for the final necessary skills areas; this included reviewing the thematic analysis that underpinned this decision making.

## Results and discussion

### Demographics of the respondents

There were 495 respondents from the 550 nurses invited to participate (90.54 %). The population was well represented by disciplines within nursing with 28.05 % being academics (*n* = 139); 24.84 % clinicians (*n* = 123); 20.2 % educators working with health services (*n* = 100) and 10.91 % Directors or Assistant Directors of Nursing (*n* = 54). Of the academics, most reported being at Lecturer/Senior Lecturer level (79 %; *n* = 108). Of note a significant number of respondents (118 representing 37 %) reporting having more than one role. This is a reflection of the Australian nursing and healthcare system with many nurses having more than one job or role.

In terms of ‘duration in role’; there were 34.8 % (*n* = 133) having been in their current role up to 2 years; 39.7 % (*n* = 151) over 6 years and slightly lesser proportion in the 3 to 5 year category 25.5 % (*n* = 97). Interestingly this was the least well answered question only 381 of the possible 495 responded (76.97 %).

State and territory representation as well as geographical locations were important to ensure that there was wide ranging inclusive consultation. There were 5 respondents from outside Australia and 15.7 % of respondents (*n* = 78) worked in rural or remote locations in Australia. Only a minority (*n* = 51; 10.4 %) of respondents reported that they were from a ‘Culturally or Linguistically Diverse’ (CALD) background.

The range of clinical settings included 10 % (*n* = 49) from Aged Care; 14.4 % from Mental Health (*n* = 71) and 5.4 % from both community and primary care settings (*n* = 27) with the largest proportion from acute care settings (around 65–70 % of the clinicians). These figures are difficult to fully interpret, as nurses in Australia are comprehensively prepared; many respondents identified that they worked in a range of practice settings, a significant number in two or more settings. Graduate programme managers and ongoing professional development involved in education totalled 11 %. Primarily 15 % (*n* = 75) of respondents worked in the private sector with 85 % (*n* = 421) in the public sector. This is an important acknowledgment that the population/cohort represents the main professional practice areas – unlike previous studies in this area were respondents often represented only acute tertiary settings (e.g. Boxer and Kluge 2000 [12]).

The majority of respondents had completed hospital based initial registration qualifications (56 %, *n* = 277), with less than a third completing an initial registration degree. Nearly all respondents; (83 %; *n* = 411) reported that they held a qualification beyond that their initial nursing degree.

### Modified Delphi rounds

In Round One of the Delphi survey respondents were asked if the skills area was ‘necessary’ or ‘not necessary’ for a newly graduating registered nurse. Of 550 invitations to the survey 495 responded (*n* = 495; 90 %). Table 1 illustrates the findings in terms of the respondent’s view that a ‘skill area’ was necessary. Two thirds of the skills areas (*n* = 20; 67.7 %) were ranked as necessary by 90 % or more of respondents (*n* = 446) and the top 25 met the 80 % threshold criteria for inclusion in round 2 of the Delphi. That is between 408 (82.4 %) and 490 (99 %) respondents believed the top 25 were ‘necessary’ skills areas for a new graduate RN.

**Table 1:** The ranked list of skills areas as identified by respondents

Ranking	Skill area	Necessary
1	Communication and documentation.	99.0 %
2	Privacy and dignity.	98.8 %
3	Efficient and effective communication.	98.6 %
4	Professional nursing behaviours - includes collaborative approaches to care.	98.4 %
5	Medications and IV products.	98.2 %
6	Teamwork and multidisciplinary team working.	98.0 %
7	Planning of nursing care.	97.8 %
8	Personal care – ability to assess, plan, implement and evaluate care of clients across a range of settings using a holistic, comprehensive nursing model.	97.6 %
9	Knowledge of key nursing implications of common medical/surgical patient presentations	97.6 %
10	Cultural competence.	97.1 %
11	Clinical intervention; preparing, assisting after care (investigations/surgery/diagnostic).	95.9 %
12	Preventing risk and promoting safety – duty of care.	95.7 %
13	Clinical monitoring and management - Use of assessment tools.	95.5 %
14	Therapeutic nursing behaviours/respectful of personal space.	94.7 %
15	Critical analysis and reflective thinking.	93.9 %
16	Dealing with emotional and bereaved people.	92.6 %
17	Learner/evidence based practitioner.	92.0 %
18	Demonstrates behaviour conducive to learning.	90.6 %
19	Promotes self-care.	90.4 %
20	Dementia related skills.	90.0 %
21	Learning and developmental culture – learning environment.	89.6 %
22	Mental health nursing care.	88.8 %
23	Coordinating skills regarding nursing process – uses a range of appropriate assessment strategies and skills across a range of settings.	88.3 %
24	Understanding the different roles of RNs in different treatment or care settings.	85.7 %
25	Technology and Informatics.	82.4 %
26	Demonstrates teaching/educator skills.	68.3 %
27	Acts as a resource.	67.5 %
28	Case manager.	58.3 %
29	Supervisory skills.	57.5 %
30	Leadership skills.	38.1 %

There is clear evidence that communication and professional behaviour; the ‘human factors’ components are ‘necessary’, as are privacy and dignity and managing medication administration. These are areas where new graduate RNs are perceived as not work ready (Burns & Poster 2008 [3]; Smith & Crawford 2003 [4]) yet the respondents suggest these are ‘necessary’.

There is less than 2 % differentiating the top ranked ten skills areas; a significant number of comments from respondents illustrate the need for good, efficient and effective communication skills; an example of a comment;

“*….is effective communication skills. If the nurse, whether student, new graduate, or other are unable to understand and communicate(with) patients and peers, the entire system breaks down and they are not able to learn/implement any care.*”

This was also linked to professional behaviours by respondents;

“*…. I would like to see more about the higher level communication skills needed to present information at handovers, case reviews and ward rounds. Documentation in progress notes is often poorly done (sic)….*”

Such comments from respondents illustrate the interconnectedness of the skills and competencies that make up nursing; in essence the art and science of nursing practice. Particular comments also centred on the notion of critical thinking; this related to how new graduate RNs and experienced students would be ‘expected to’ review any clients condition in any setting and be able to consider the need to escalate care through additional forms of focused assessment.

Respondents were also invited to add any skills areas that were not listed in the survey from the initial thirty identified from the Brown et al 2015 [1] audit of skills. A number of additional skills areas were highlighted by respondents;

*‘perioperative nursing skills’, ‘business planning’, ‘ensuring staff are competent’, ‘de-escalation skills’, ‘capacity to undertake action research’, ‘capacity to precept students’, ‘instil the theory of education/teaching’ and ‘awareness of the cost of health care’, ‘health literacy’*.

These were only identified by one or two respondents and so they were not included in the next round due to insufficient respondent support.

Finally there were a greater number of comments focussing on the lower ranked skills areas, why particular skills were deemed not necessary and a commonly held view that the new graduate RN needed time to consolidate their learning and skills.

Examples were about the new graduate not being a;

‘*mature educator’; ‘mature researcher’; ‘competent user of research findings’; ‘leader on graduation but developing’; ‘mature leader/case manager/supervisor*’

A key theme from the respondents were that new graduate RNs are continuing to develop these five skills areas; Leadership, Supervisory skills, Case Manager, Acts as a resource and Demonstrates Teacher/Educator skills. Many respondents stated that the new graduate had learned a great deal from their nursing studies and they are continuing to develop in these areas as they consolidate their learning.

These responses clearly identify that the new graduate RN needs some level of leadership and supervisory skills at the point of registration, further research in this area is needed to clarify what form that education might take and how it may be embedded in pre-registration nursing programmes (Brown et al 2015 [46]).

In round 2 of the modified Delphi the respondents were asked if they agreed with the final skills list. All thirty skills areas were retained and of the 495 initial respondents 299 agreed with the skills areas list from the 322 responders (93 %). No additional skills areas were suggested to be added or removed.

## Conclusion

The findings from this study have identified that the thirty skills areas are perceived to be ‘necessary’ by respondents who represented a range of academics and clinicians from diverse clinical settings. New graduate RNs from comprehensively prepared nursing degree programmes in Australia are able to find employment in a range of clinical areas and so these findings have a bearing on the skills that should be taught within nursing programmes in Australia in order to meet the identified skills set of a new graduate RN.

This work articulates the skills areas by identifying the work of the nurse and providing a context for that action, for example the skills area “*Clinical monitoring and management - Use of assessment tools all forms of assessment are included here”* is a skill area that is highly ranked (by over 95 % of respondents) as necessary for the new graduate RN. Within this skill area there are aspects of critical thinking in which an RN is expected to review the client or patient in any setting through observation, and in that review escalate if necessary the level of assessment from observing and noticing to the instigating the use of a particular assessment tool. It is expected within this that the RN will communicate the urgency of the situation, if necessary, to others. These actions are appropriate in any clinical setting and could be undertaken within a community, a mental health or an acute health care setting.

Earlier works in the area (Wolff et al 2010 [47], Danbjørg and Birkelund 2011 [48]) illustrate the overly reductionist approach citing ‘hygiene skills’ or ‘central venous line management skills’. The perceptions of newly graduating RN’s illustrates their wish to be able to perform as many of the skills as possible but realising that they will need to learn the art of nursing as they work in the role of the registered nurse in whichever practice setting they find themselves located (Danbjørg and Birkelund 2011 [48]).

There are implications for nursing education; the duration of the course of study is an important area to consider. The amount of time is limited within any academic and practice based programme and so curriculum design as well as appropriate pedagogy need to reflect clear empirically derived content. If every item that was raised was to be added to the programme then the course of study would be significantly and unreasonable increased. The thirty skills areas reflect the complexity and artistry of nursing practice without reducing nursing to a list of skills. This in tandem with the numerous comments indicating that students, as they progress through an eligibility to practice programme, need to prepare and develop skills that will provide a foundation for future learning and practice is critical to the new graduate RN.

There are also implications for practice; suggesting that student nurses need to be given greater opportunities within their programmes of study to explore and practice in a safe and supportive environment the thirty skills areas in order to better prepare them for practice.

### Relevance to practice

Nursing is a practice based discipline and as such the skills that nurses need to demonstrate in their practice require careful definition and clarification so that both the student and then the assessor in practice and simulation can be clear on what is expected of an RN. Finally the client and patients and their families also need to be aware of what they can expect of a qualified RN in practice – this work begins that exploration.

### Limitations of the study

This study based the modified Delphi survey on what was claimed to be taught in pre-registration nursing programmes in Australia rather than invite respondents through the survey to identify what they thought was appropriate – this may have influenced the respondents. Secondly nursing is such a practical profession, and as here in Australia, nurses are comprehensively prepared the drive towards the competency based framework may lead respondents to believe that ‘skills’ alone are not important – this work begins to explore the connection between skills and competence and as such need further exploration.

The complex participant population in which nurses have more than one role makes it difficult to clearly articulate data around clinical backgrounds. This population reflected that diversity but there were still a relatively high population of acute care nurses.

Respondents, as one would expect, reflected their own clinical background and interests. This however did not detract from what could be reasonably expected of a comprehensively prepared new graduate nurse in practice.

### Ethics and consent

Ethical approval was sought and granted from the University of Wollongong Human Research Ethics Committee (Approval no. HE08/142).

### Consent to publish

Not Applicable.

### Availability of data and materials

Detailed data will not be shared due to the confidential nature of the way that the data has been managed.

## Abbreviations

ALTC: Australian learning and teaching council
ANMC: Australian nursing and midwifery council
ANMAC: Australian nursing and midwifery accreditation council
CDNM: Council of deans of nursing and midwifery
I.V. or I.V.I.: Intravenous infusion
MMSE: Mini mental state examination
NMBA: Nursing and midwifery board of Australia
OLT: Office of teaching and learning
RN: Registered nurse
RUDAS: Rowland universal dementia assessment scale
